# The health and condition responses of Delta Smelt to fasting: A time series experiment

**DOI:** 10.1371/journal.pone.0239358

**Published:** 2020-09-24

**Authors:** Bruce G. Hammock, Wilson F. Ramírez-Duarte, Pedro Alejandro Triana Garcia, Andrew A. Schultz, Leonie I. Avendano, Tien-Chieh Hung, James R. White, Yih-Tyng Bong, Swee J. Teh

**Affiliations:** 1 Aquatic Health Program, School of Veterinary Medicine, Department of Anatomy, Physiology, and Cell Biology, University of California, Davis, California, United States of America; 2 Grupo de Investigación en Sanidad de Organismos Acuáticos, Instituto de Acuicultura de los Llanos, Universidad de los Llanos, Villavicencio, Meta, Colombia; 3 Science Division, U.S. Bureau of Reclamation Bay-Delta Office, Sacramento, CA, United States of America; 4 Fish Conservation and Culture Laboratory, Biological and Agricultural Engineering Department, University of California, Davis, Davis, CA, United States of America; 5 California Department of Fish and Wildlife, Stockton, CA, United States of America; Kafrelsheikh University, EGYPT

## Abstract

There is an extensive literature establishing, validating, and quantifying a wide range of responses of fishes to fasting. Our study complements this work by comparing fed and unfed treatments of hatchery-raised Delta Smelt (*Hypomesus transpacificus*)—an imperiled fish that is endemic to the San Francisco Estuary and its tributaries in California, USA—across a diverse suite of endpoints over a two-month time series. The experiment was conducted at 15.9°C, and individuals were sampled at 12 time points as starvation became increasingly severe. We found that hepatosomatic index and condition factor were relatively sensitive to starvation, becoming significantly depressed at Day 4 and 7, respectively. Histological analysis of liver showed elevated cytoplasmic inclusion bodies at Day 7, followed by increased glycogen depletion, single cell necrosis, and hydropic vacuolar degeneration at Day 14, 21, and 28, respectively. Of four antioxidants measured, glutathione decreased at Day 4, superoxide dismutase increased at Day 14, catalase increased at Day 56, and glutathione peroxidase was not affected by starvation. The net result was a ~2-fold increase in lipid peroxidation (malondialdehyde) in fasted fish that was highly inconsistent through time. RNA to DNA ratio and triglycerides in muscle were relatively insensitive to starvation, only consistently decreasing with fasting after mortality began increasing in the ‘No Feeding’ treatment, at Day 21. Together, these results suggest that Delta Smelt mobilize hepatic energy stores far more rapidly than lipids in muscle when subjected to fasting, leading to rapid atrophy of liver and the development of cytoplasmic inclusion bodies—possibly autophagosomes—in hepatocytes.

## Introduction

A major consequence of anthropogenic global change is the disruption of aquatic food webs. Moderate nutrient increases, for example, can stimulate primary productivity, leading to elevated fishery production [1]. Higher levels of nutrients may lead to excess primary productivity, hypoxia, and fish kills (e.g., Gulf of Mexico, Mediterranean Sea, Black Sea; [1]). Invasive species can be particularly disruptive to aquatic food webs, with effects ranging from prey field shifts to trophic cascades (e.g., [2, 3]). Given the preponderance of ectotherms, aquatic food webs are also highly vulnerable to climate change [4–6]. As the climate warms, the oxygen and feeding requirements of ectotherms increases, even as dissolved oxygen decreases [6, 7]. These changes can also interact. For instance, high temperatures and nutrients can trigger blooms of the invasive cyanobacteria *Microcystis* spp. When *Microcystis* spp. cells lyse, communities are exposed to microcystin, a potent hepatotoxin that bioaccumulates in aquatic organisms [8–12]. Thus, given the global alterations of aquatic food webs, understanding how aquatic organisms respond is key.

Commonly used tools for identifying altered food web linkages are metrics of nutritional status in fish. These metrics often include Fulton’s condition factor, hepatosomatic index, RNA to DNA ratio in muscle, triglyceride concentration in muscle, and liver glycogen. Condition factor reflects nutritional status because body mass responds more quickly to food limitation than body length (e.g., [13]). Similarly, hepatosomatic index is responsive to food limitation because liver weight declines more rapidly than body weight as hepatic energy stores are rapidly mobilized (i.e, glycogen, lipid, and protein; [14–16]). The ratio of RNA to DNA in muscle is an indicator of recent growth, and therefore fasting, because RNA concentration in cells correlates with protein synthesis, whereas DNA concentration remains constant (e.g., [17, 18]). Triglycerides, the main long-term energy substrate in fishes, are also widely used, and tend to reflect longer-term responses to food limitation [19, 20]. However, the speed and extent to which these metrics respond to changes in diet depend on the study organism, experimental conditions, and reproductive stage [21]. For example, while liver glycogen depletion typically declines rapidly in response to food limitation (e.g., [15, 22]), Nagai and Ikeda [23] reported no decrease in liver glycogen in carp (*Cyprinus carpio*) following 22 days of fasting, Sockeye Salmon migrate 1000 km with no decrease in liver glycogen, and hepatic lipid and glycogen were mobilized simultaneously during early fasting in Atlantic Cod [21, 24, 25]. Thus, species specific laboratory validation is helpful for interpreting biomarkers of nutritional stress.

Given that the liver is highly sensitive to starvation, its condition is especially important for understanding nutritional stress in fishes. Histology studies indicate that the initial sensitivity of liver weight to fasting is due to a rapid atrophy of hepatocytes as energy sources are consumed (e.g., [16, 26, 27]), rather than because of a decline in the number of hepatocytes [28]. For example, Fernández-Díaz et al. [29] noted several alterations in the liver of *Solea senegalensis* larvae fed inert diet, including a decline in hepatocyte size, the loss of lipid vacuoles, and an increase in basophilia, possibly related to reduced protein synthesis. At later stages of fasting (i.e., starvation), hepatocyte loss occurs via several interrelated cell death mechanisms, including necrosis and apoptosis [30].

Another potential hepatic response to starvation is oxidative damage, which occurs when the production of reactive oxygen species overcomes antioxidant defense mechanisms [31]. The liver is the main organ for metabolic control, plays a key role in production of reactive oxygen species, and is therefore vulnerable to oxidative damage [32]. Major antioxidant enzymes include superoxide dismutase, catalase, and glutathione peroxidase [33], while glutathione is the main non-enzymatic antioxidant [34]. Glutathione is the co-substrate for glutathione peroxidase and glutathione S-transferase and is the most abundant low molecular weight thiol synthesized by cells [34]. One of the major mechanisms of cellular injury due to oxidative damage is lipid peroxidation, typically indicated by elevated levels of the lipid peroxidation product malondialdehyde [35]. However, the oxidative stress response to fasting in fish livers is difficult to predict. On one hand, food is necessary for the acquisition and production of antioxidants, so fasting might be expected to increase oxidative stress [36]. On the other hand, metabolic demand of fishes declines rapidly with food deprivation [16, 37], which reduces the production of free radicals as the rate of mitochondrial respiration declines [38]. Studies to date suggest that glutathione and antioxidant enzymes in the livers of fishes are reduced by starvation, leading to oxidative stress [32, 33, 39].

Here, we conducted a time series experiment comparing the sensitivities of a wide range of biomarkers along a gradient of mild to severe starvation. We define sensitivity as the length of time (a proxy for severity of starvation) before a statistically significant difference was detected, with earlier detection considered more sensitive. We conducted the study on hatchery-raised Delta Smelt (*Hypomesus transpacificus*), a species listed as threatened and endangered at the federal and state levels, respectively [40]. Delta Smelt is endemic to the Sacramento and San Joaquin River Delta and San Francisco Estuary (SFE), an ecosystem that exhibits chronically low pelagic productivity [41–43]. There is a history of biomarker and stomach fullness work on Delta Smelt collected from the field, which provides evidence of contaminant and food limitation stress in the species [44, 45]. However, the nutritional biomarkers have not been empirically validated in Delta Smelt, causing difficulties both in understanding the sensitivities of biomarkers to nutritional stress, and distinguishing between liver abnormalities caused by poor nutrition or contaminants, another known stressor in the SFE [46–48]. This study will therefore improve interpretation of health and condition data collected from wild Delta Smelt, informing conservation and restoration efforts. Key features of the study are that we 1) sampled up to 12 time points (depending on the biomarker), particularly early in the experiment, and 2) quantified a wide range of endpoints, including condition and hepatosomatic indices, biochemical, immunohistochemical, and histological biomarkers of nutritional stress, as well as daily mortality. These study features allowed us to compare the sensitivities of the biomarkers, gain a more mechanistic understanding of the progression of starvation in Delta Smelt and fish more generally, and to associate specific liver abnormalities with poor nutrition. Finally, we compared the starved and fed hatchery-origin experimental Delta Smelt to wild Delta Smelt in terms of the nutritional metrics examined in both groups.

## Methods

### Experimental endpoints

We examined six indices in the fasting experiment that we also measured on wild Delta Smelt. Fulton’s condition factor, hepatosomatic index, RNA to DNA ratio in muscle, and triglyceride concentration in muscle were measured at 12 time points (Table 1). Liver lesion score (a summation of liver lesions scored histologically), and liver glycogen depletion (also scored histologically) were assessed at 10 time points (Table 1). The liver histopathology scoring and lesions are described in detail in Teh et al. [49]. As a summation of the severity scores of liver lesions, liver lesion score provided a general indication of liver condition (higher scores indicate worse condition, [44, 49]; individual lesions in Table 2). The responses of the disaggregated lesions are also provided (Table 2). We expected liver lesion score to increase as starvation progressed as specific lesions were triggered and exacerbated by starvation. For example, we expected single cell necrosis or apoptosis to occur as hepatocytes starved, and then macrophages to aggregate around the dead cells [50].

**Table 1:** Sampling time points, details, and effort for each endpoint examined in the study. Preservation is the initial method used to preserve the fish, ‘n’ refers the number of fish per time-point (i.e., for condition factor 16 fish were used from each treatment), and ‘Wild’ refers to whether the endpoint was also measured on wild fish.

Endpoint	Method	Tissue	Time points (days)	n	Preservation	Wild
Condition factor	Morpho/Grav	Fish	0, 1, 2, 4, 7, 14, 21, 28, 35, 42, 49, 56	32	L nitrogen	Yes
Hepatosomatic index	Gravimetric	Liver/fish	0, 1, 2, 4, 7, 14, 21, 28, 35, 42, 49, 56	32	L nitrogen	Yes
RNA/DNA	Biochemical	Muscle	0, 1, 2, 4, 7, 14, 21, 28, 35, 42, 49, 56	32	L nitrogen	Yes
Triglycerides	Biochemical	Muscle	0, 1, 2, 4, 7, 14, 21, 28, 35, 42, 49, 56	32	L nitrogen	Yes
Lesion score	Histological	Liver	0, 4, 7, 14, 21, 28, 35, 42, 49, 56	8	Formalin	Yes
Glycogen depletion	Histological	Liver	0, 4, 7, 14, 21, 28, 35, 42, 49, 56	8	Formalin	Yes
Hepatocyte area	Histological	Liver	0, 4, 7, 14, 24, 28	8	Formalin	No
Hepatocyte nucleus area	Histological	Liver	0, 4, 7, 14, 21, 28, 35, 42, 49, 56	8	Formalin	No
Apoptosis	Immunohisto	Liver	0, 24, 42, 56	1	Formalin	No
Glutathione	Biochemical	Liver	0, 1, 2, 4, 7, 14, 21, 28, 35, 42, 49, 56	8	L nitrogen	No
Malondialdehyde	Biochemical	Liver	0, 1, 2, 4, 7, 14, 21, 28, 35, 42, 49, 56	8	L nitrogen	No
Superoxide dismutase	Biochemical	Liver	0, 7, 14, 21, 56	8	L nitrogen	No
Catalase	Biochemical	Liver	0, 7, 14, 21, 56	8	L nitrogen	No
Glutathione peroxidase	Biochemical	Liver	0, 7, 14, 21, 56	8	L nitrogen	No
Protein concentration	Biochemical	Liver	0, 7, 14, 21, 56	8	L nitrogen	No
Mortality	Count	Fish	Daily throughout 2 month experiment	NA	NA	No

Abbreviations: Immunohisto: immunohistochemistry, Morpho: morphometric, Grav: gravimetric, L nitrogen: liquid nitrogen.

**Table 2:** Mean liver histological results from the experiment.

	GD		HVD		SCN		MA		INF		CIB		LIP	
Day	F	No F	F	No F	F	No F	F	No F	F	No F	F	No F	F	No F
0	1.3	0.5			0.25					0.25				
4	0.8	0.7			0.25	0.67						0.33		
7	1	1			0.5						0.25	0.75		
14	1	2.5				0.5		0.75				0.75		
21	0.5	2				1			0.25	0.25		0.75		
28	0.5	1		0.5				0.25		0.25	0.25			
35		2.5		0.75		0.75				0.25		0.25	0.25	
42	0.5	2.8		0.75		2.25		0.5					0.25	
49	1	2.8		1.25		0.75	0.25	0.5					0.25	
56		1.5		1		1.25		0.25		0.25				

F is Feeding, No F is No Feeding. GD is glycogen depletion, HVD is hydropic vacuolar degeneration, SCN is single cell necrosis, MA is macrophage aggregate, INF is inflammation, CIB is cytoplasmic inclusion body, and LIP is lipidosis. 0 = absent/minimal, 1 = mild, 2 = moderate, and 3 = severe. Note that fields with mean scores of 0.0 were left blank.

We included ten additional endpoints that we do not routinely measure on wild fish (Table 1). Mortality was recorded daily. Hepatocyte area was measured at six time points, hepatocyte nucleus area at ten time points, and apoptosis at four time points (Table 1). These endpoints were included to understand whether livers of fasting fish decline in size due to atrophy of hepatocytes, loss of cells via apoptosis or necrosis, or some combination of these processes. Glutathione and lipid peroxidation (indicated by malondialdehyde) were both measured in liver at 12 time points. The enzymatic antioxidants superoxide dismutase, catalase, and glutathione peroxidase, as well as protein concentration, were measured in liver at five time points. Measurement of the oxidative stress endpoints allowed us to evaluate the impact of starvation on antioxidants, cell/tissue damage and the potential implications for fish health [31]. We expected starvation to deplete the concentration of total glutathione, tipping the balance toward a prooxidant state, enhancing hepatic lipid peroxidation. Decisions on how many time-points to sample and whether to pool tissue were based on maximizing use of the limited quantity of tissue available, feasibility (hepatocyte area, explained below) and cost, to a lesser extent.

### Food limitation experiment

We conducted the fasting experiment at the Fish Conservation and Culture Laboratory (FCCL), near Byron, CA, USA [51]. The FCCL raises Delta Smelt in bead-filtered, UV-treated, recirculated, and temperature-controlled water pumped from the Sacramento-San Joaquin Delta. A two-week acclimation period began on Sept 24, 2018 when 800 Delta Smelt (143 days post-hatch) were moved from two holding tanks into eight experimental tanks in black ‘5-gallon’ buckets, at a density of 100 fish tank^-1^ (S1 Fig). At the time of sampling, mean fork length and body weight was 45.7 mm (SD = 6.4) and 0.54 g (SD = 0.27), respectively. Sub-adults were used in the experiment to both minimize the influence of reproduction on liver condition of female fish (glycogen depletion, [49]), while also providing sufficient tissue for each endpoint. The fish were in the buckets for no more than five min during transfer to the tanks. Tanks were roughly cylindrical, black, and plastic (S1 Fig). Tank depth was 60 cm, wetted depth was 47 cm, diameter was 100 cm, and the working volume was 290 L. The fish in all eight tanks were fed to satiation throughout the acclimation period (i.e., aiming for a food to feces ratio of 0.25 on the bottom of the tanks), using the same feed on which they were cultured (Bio-vita Crum #1). Feedings occurred five times during the day (at ~06:45, 08:00, 10:00, 12:00, and 14:00). The tank bottoms were cleaned with a siphon three days per week, and this effort was maintained for all tanks throughout the experiment. We installed 5-μm filters on the inputs to each tank to ensure that no food particles entered the tanks other than during feedings (‘10 inch by 2.5-inch sediment filters’, iSpring Water Systems, LLC, Alpharetta, Georgia; S1 Fig). The handful of mortalities that occurred during the two-week acclimation period were replaced with fish from the same cohort so that the density was exactly 100 fish tank^-1^ at the start of the experiment.

The experiment had two treatments called ‘Feeding’ (i.e., control) and ‘No Feeding,’ which were randomly assigned to the eight tanks (four tanks per treatment). We continued feeding the Feeding treatment according to the regime described above. The final feeding for the No Feeding treatment occurred on Oct 8 at 08:00 (Day 0), and no further food was provided for the remainder of the eight-week experiment. Fish were sampled from ~10:30–12:30 at Day 0, 1, 2, 4, 7, 14, 21, 28, 35, 42, 49, and 56. At all but the final time point, four fish per tank were removed from the tanks with an aquarium net, gently shaken to remove water, wrapped in aluminum foil, and flash-frozen in liquid nitrogen [52]. At the final time point, one of the No Feeding tanks had only two fish remaining, which were both flash-frozen (all other tanks had at least 4 fish remaining). In addition to the flash-frozen samples, an additional fish from each tank was sampled, anesthetized in an ice-water bath, and placed in 10% buffered formalin for fixation and subsequent histology at a subset of the time points (Day 0, 4, 7, 14, 21, 28, 35, 42, 49, and 56).

Individual fish were not tagged, so initial measurements of length and weight were not made. While these measurements would have improved the sensitivity of condition factor and hepatosomatic index by accounting for inter-individual variation, they would have stressed the fish, and Delta Smelt is an unusually sensitive species [53]. In addition, we wanted the results to mimic the sensitivity of our long-term study on Delta Smelt in the wild, in which repeated measurements on the same individual cannot be made.

Water temperature was measured hourly throughout the experiment (HOBO Water Temp Pro v2, Onset). Ammonia, nitrite, and nitrate levels were measured using Hach testing kits every 3–4 days (Loveland, CO). Dissolved oxygen, pH, and salinity were also measured every 3–4 days, using a handheld YSI-85 m (YSI Incorporated, Yellow Springs, OH, USA). Mortalities were recorded and removed daily (only live sampled fish were measured for biomarkers).

### Ethics statement

This study was approved by the University of California, Davis Institutional Animal Care and Use Committee and followed the experimental protocol for Animal Care and Use protocol #19872. The predefined humane endpoint was swimming activity, with our protocol stipulating that the experiment would be terminated when overall differences in swimming activity between the treatments were observed during daily monitoring. Fish in both treatments remained active throughout the experiment, so this criterion was not met. Mortality was not a planned outcome, but mortalities were recorded as part of standard protocol at the hatchery, so the data were included post-hoc. Occasional fish that appeared to be under duress in either treatment were not euthanized because it was difficult to predict whether such small, delicate fish would recover. Delta Smelt exhibit high mortality rates even under optimal conditions [51]. All staff obtained animal care and use training from UC Davis.

### Laboratory fish processing

The 382 flash-frozen Delta Smelt sampled during the experiment were dissected at UC Davis following a slightly modified version of the methods of Teh et al. [52]. Briefly, each fish was removed from liquid nitrogen, photographed, weighed on an analytical balance (±0.01 mg), and measured for fork length. Each fish was dissected as it thawed over 5–10 min. Dorsal muscle tissue was excised, weighed on an analytical balance (±0.1 mg), frozen in liquid nitrogen, and then stored at -80°C until processing for RNA to DNA ratio and triglycerides. The livers were removed from each fish, weighed, pooled in a 1.5-mL tube (4 livers per tube), placed in liquid nitrogen, and stored at -80°C for oxidative stress analysis (glutathione, superoxide dismutase, catalase, glutathione peroxidase, and malondialdehyde). Three fish were excluded because they had livers that were too small to be excised.

Fulton’s condition factor and hepatosomatic index were calculated as follows: condition factor = (W_B_ / FL^3^) × 100 and hepatosomatic index = (W_L_ / W_B_) × 100, where W_B_ is the body weight (mg), FL is the fork length (mm) and W_L_ is the liver weight in mg [54].

Triglyceride concentration was measured in the frozen dorsal muscle samples using an adipogenesis assay kit (Catalog #K610- 100, Biovision, CA, USA), as per the manufacturer’s instructions, and standardized to protein concentration using the method of Lowry et al. [55]. Samples were analyzed using a microplate reader (Tecan Infinite M200, Männedorf, Switzerland). Triglyceride concentration is reported in μmol of triglyceride per mg of protein.

RNA to DNA ratio in dorsal skeletal muscle was measured using the ethidium bromide fluorometric technique reported by Caldarone et al. [56].

Activities of catalase, superoxide dismutase, and glutathione peroxidase as well as lipid peroxidation by the thiobarbituric acid reactive substance (TBARS) assay were assessed in pools of 4 livers per tank-time point combination as described in Ramírez-Duarte et al. [57] with some modifications. Livers were homogenized by grinding at 4°C in 50 mM potassium phosphate buffer (pH 7.4) containing 1 mM EDTA, 1 mM phenylmethanesulfonyl fluoride, 1% peroxidase free Triton X-100 and 1 mM dithiothreitol using a liver weight:buffer volume ratio of 1:20. Raw homogenate was used to assess lipid peroxidation following the method described by Ohkawa et al. [58] with some modifications and reported as malondialdehyde in nmol/g wet tissue. Homogenates were centrifuged at 10,000 × g for 20 min at 4°C and the resulting supernatant was stored at -80°C until analysis. Superoxide dismutase activity was determined by measuring the combined effects of EDTA, manganese ions and mercaptoethanol on the rate of NADH oxidation according to Paoletti et al. [59]. Briefly, a final volume of 200 μL of reaction cocktail containing 0.05 mM potassium phosphate buffer, 0.25 mM NADH, 3.5 mM EDTA, 1.75 mM MnSO_4_, 4 mM mercaptoethanol, and 5 μL of supernatant was prepared and absorbance at 340 nm was measured for 20 min at 25°C. Superoxide dismutase activity is reported as Units/mg protein in the supernatant, where one unit is equal to 50% inhibition of NADH oxidation of the blank. The catalase assay was performed as described by Fernández-Díaz et al. [29] by following the reduction in absorbance of hydrogen peroxide at 240 nm in 96-well plates. Catalase activity was reported as Units/min/mg protein. Glutathione peroxidase activity was assessed by recording the oxidation of NADPH at 340 nm according to the method of Flohé and Günzler [60] and reported as Units/min/mg protein. A microplate reader (Tecan Infinite M200, Männedorf, Switzerland) was used to measure changes in absorbance. Protein concentration in the supernatant was measured following Lowry et al. [55] using the DC protein assay kit (Bio-Rad Laboratories, Hercules, CA, USA) and reported as mg/mg wet tissue. Concentration of total glutathione was measured as described in Ramírez-Duarte et al. [57] and reported as nmol/mg wet tissue.

The fish that were fixed directly in formalin were used for histological analysis (Day 0, 4, 7, 14, 21, 28, 35, 42, 49, and 56). Four transverse sections along the length of each fish were made using a razor blade (~4 mm thickness). Sections were taken immediately behind the opercular cavity, between the opercular cavity and the dorsal fin, just anterior to the dorsal fin, and just posterior to the dorsal fin. The four transverse sections were processed for histology and stained either with eosin and hematoxylin for histopathological assessment or periodic acid-Schiff (PAS) for glycogen depletion assessment following Teh et al. [61].

Using a compound microscope, the liver slides were scored for seven characteristics: glycogen depletion, hydropic vacuolar degeneration, single cell necrosis, macrophage aggregate, inflammation, cytoplasmic inclusion bodies, and lipidosis. Hydropic vacuolar degeneration is typified by a single large, hydropic-appearing vacuole, with smooth edges that nearly fills affected cells, with occasional unknown material within the vacuole [62]. The other histologic endpoints are described in Teh et al. [49]. Each endpoint was scored on an ordinal scale of 0–3, where 0 = absent/minimal, 1 = mild, 2 = moderate, and 3 = severe. Thus, the higher the score the more damaged or glycogen depleted the liver. The scores for each lesion (all but glycogen depletion) were summed to produce a composite score indicative of the overall condition of the liver (i.e., ‘lesion score’; [44, 49]). Liver glycogen depletion was analyzed separately because it is not a lesion, and it typically responds within days to one week in response to food deprivation, whereas we expected the other endpoints to respond more slowly [15, 22, 63]. The liver was also screened for parasites, bacterial infection, preneoplastic foci and neoplasms, but these alterations were not observed.

**Fig 1:**
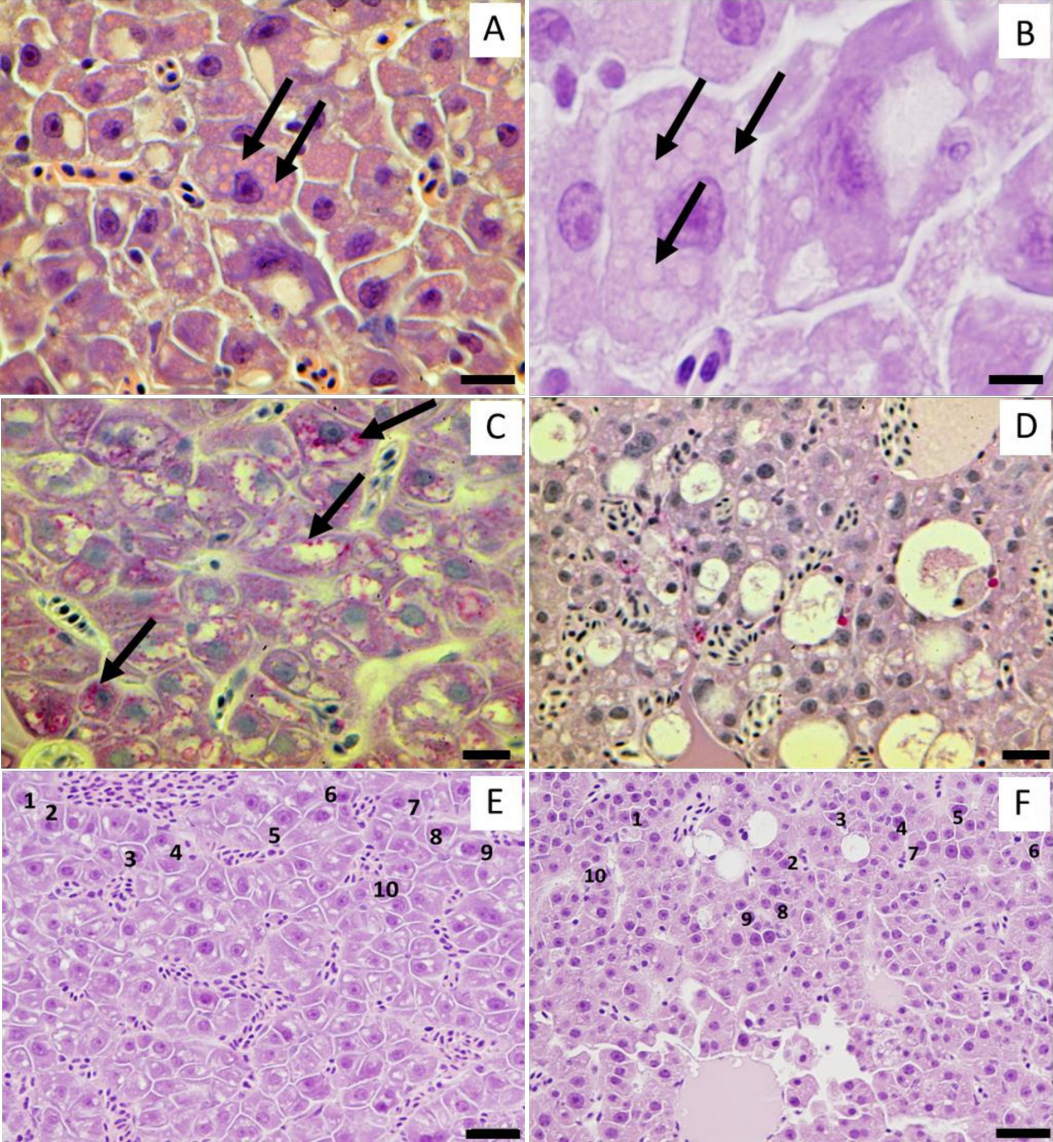
Liver histopathology. Panel A: Liver of Delta Smelt from the No Feeding treatment at Day 4. Arrows indicate eosinophilic cytoplasmic inclusion bodies, possibly autophagosomes. Panel B: Liver of Delta Smelt from the No Feeding treatment at Day 7, cytoplasmic inclusion bodies (indicated by arrows) at higher magnification. Note possible double membranes. Panel C: Liver of Delta Smelt from the Feeding treatment, PAS stain. Arrows indicate glycogen granules in the cytoplasm of hepatocytes that are stained magenta (compare to panel D). Panel D: Liver from Delta Smelt from the No Feeding treatment at Day 21, PAS stain. No magenta glycogen granules observed in cytoplasm of hepatocytes (compare to panel C). Panel E: Liver of Delta Smelt from the Feeding treatment, H&E stain; the numbered hepatocytes were used to measure hepatocyte area. Panel F: Liver of Delta Smelt at Day 21 from the No Feeding treatment, H&E stain. Note atrophied hepatocytes (compare to Panel E). For both the Feeding and No Feeding treatments, hepatocytes were selected for area measurement based on three criteria: 1) well demarcated cell membranes, 2) presence of nucleolus in the nucleus, and 3) cells were along a ‘backward S’ path moving from the top-left corner to the bottom-right corner of each photo (Panels E and F). Scale bars = 20 μm (Panel A), 8 μm (Panel B), 15 μm (Panels C and D), 20 μm (Panels E and F).Using the formalin-fixed fish, liver tissue from the No Feeding treatment at Day 0, 21, 42, and 56 was prepared for immunohistochemical detection of apoptosis. Liver tissue from two fed fish was used as a control (Day 0 and 49). Tissues for immunohistochemistry were sectioned (3 μm) and adhered to Superfrost^®^Plus Microscope Slides (Fisher Scientific, Pittsburgh, PA, USA). Copper-exposed olfactory tissue from Delta Smelt was used as positive control and normal horse serum was substituted for the primary antibody as a no primary antibody control. After deparaffinization and rehydration, heat-induced epitope retrieval was performed in citrate buffer (pH 6) at 95–98°C for 10 min in a microwave (800W). Endogenous peroxidase activity was blocked using 3% H_2_O_2_ in methanol for 15 min followed by non-specific blocking with 10% normal horse serum for 15 min. Anti-cleaved caspase-3 polyclonal antibodies (Cat# G7481, Promega, Madison, WI, USA) at 1:2000 dilution were applied to the sections overnight at 4°C and subsequently incubated with HRP-conjugated secondary antibodies for 30 min at room temperature. Finally, antigen-antibody complexes were visualized with chromogen for 1 min. Sections were counterstained with Meyer’s hematoxylin for 15 sec and mounted with Krystalon™ mounting media (MilliporeSigma, Burlington, MA, USA).

To measure hepatocyte and nucleus area, photographs were taken of the liver sections on each histology slide. Identical settings and magnification were used for each photo (400×). The hepatocytes and nuclei (usually 10 of each, but as few as 6) were manually outlined using the program ImageJ and the area was calculated [64]. Hepatocytes and nuclei were selected for measurement that 1) could be clearly differentiated from one-another, and 2) had a visible nucleus (for hepatocyte area measurements) or nucleolus (for nuclei area measurements), indicating that a significant proportion of the cell or nucleus volume was sectioned (Fig 1E and 1F). To prevent bias, we began measuring hepatocytes and nuclei in the upper left-hand corner of each photo and proceeded back and forth, ultimately toward the lower right corner of the photo. Along this ‘backward S’ pattern we measured every hepatocyte or nuclei that met both criteria until 10 measurements were collected for each endpoint or the end of the slide was reached. Hepatocyte area was not measured after Day 28 because it became too difficult to differentiate hepatocytes in the No Feeding treatment. Nuclei area was measured for all time points.

### Comparison to wild fish

Our lab has received wild Delta Smelt collected during California Department of Fish and Wildlife and United States Fish and Wildlife trawls in 2011 and 2017, respectively. During the trawls, wild Delta Smelt were frozen in liquid nitrogen on agency boats and sent to our lab where we measured a range of indices (e.g., [44, 49, 52, 65]. The livers were scored histologically as described above. We note that while the preservation method between the experimental and wild fish was identical for condition, hepatosomatic and biochemical indices (i.e., flash freezing in liquid nitrogen), this was not the case for liver lesion score and glycogen depletion. For the wild fish, histology was performed on flash-frozen tissue, while histology was performed on tissue preserved in phosphate buffered formalin for the hatchery fish. Therefore, the results may not be perfectly comparable, although Teh et al. [52] reported no noteworthy differences between Delta Smelt liver tissue that was flash-frozen or fixed in phosphate buffered formalin. The present study includes data from wild Delta Smelt collected from Aug 2011 through Sep 2018.

### Statistical analysis

A Kaplan-Meier survival analysis [66] was conducted to compare the mortality rates between the two treatments (Feeding [n = 400] and No Feeding [n = 400]). A log rank (Mantel-Cox) test was run to determine if there were differences in the survival distribution between the two treatments. Time to significance was determined by assessing overlap of the 95% confidence intervals of the survival curves for both treatments. The analysis was run in R.

Condition factor, hepatosomatic index, RNA to DNA ratio, and triglycerides were each analyzed using identical factorial ANOVAs. These analyses had 32 fish per time point, 16 in each treatment (except for the final time point, which had 14 fish in the No Feeding treatment). ‘Tank’ was included as a random effect to account for the repeated samples taken from the same experimental unit [67]. Fixed effects included ‘day’ (as a discrete variable), ‘treatment’ (Feeding or No Feeding), and a ‘day’ by ‘treatment’ interaction. The interaction was included to account for any treatment specific changes in each endpoint as the experiment progressed from Day 0 to 56. Significant interactions between ‘day’ and ‘treatment’ were followed by ‘test slices’ at each time point to test for differences between the Feeding and No Feeding treatments (i.e., planned linear contrasts; [67]).

Glutathione, superoxide dismutase, catalase, glutathione peroxidase, protein, lipid peroxidation, malondialdehyde, hepatocyte area, and nucleus area were analyzed with nearly identical ANOVAs to the ones described above. For glutathione, superoxide dismutase, catalase, glutathione peroxidase, protein, malondialdehyde, and lipid peroxidation, the differences were that sample size was smaller because the four livers from each tank-time point combination were physically pooled during dissections, so ‘tank’ was not included in the analysis due to the pooling (n = 8 for each time point, 4 samples per treatment). In addition, malondialdehyde was log_10_-transformed because its variance increased with its mean. For superoxide dismutase, catalase, glutathione peroxidase and protein, there were fewer time points (Day 0, 7, 14, 21, and 56). For hepatocyte and nucleus area, the differences were that there were eight fish per time point in each analysis, one fish per tank-time point combination (i.e., the subset of fish that were preserved initially in buffered formalin, not flash-frozen). In addition, ‘fish’ was included as a random effect because multiple hepatocytes were measured per fish. For hepatocyte area, only Day 0, 4, 7, 14, 21, and 28 were included. Planned linear contrasts were applied following either significant interactions between ‘treatment’ and ‘day’ or a significant effect of ‘treatment.’ For all ANOVAs, consistency with their assumptions was confirmed with residual plots.

As non-parametric endpoints, glycogen depletion and liver lesion score were analyzed with Wilcoxon Rank Sum tests between the two treatments across all time points. All ANOVAs and the Wilcoxon tests were run at an alpha of 0.05 using JMP Pro 14.

**Fig 2:**
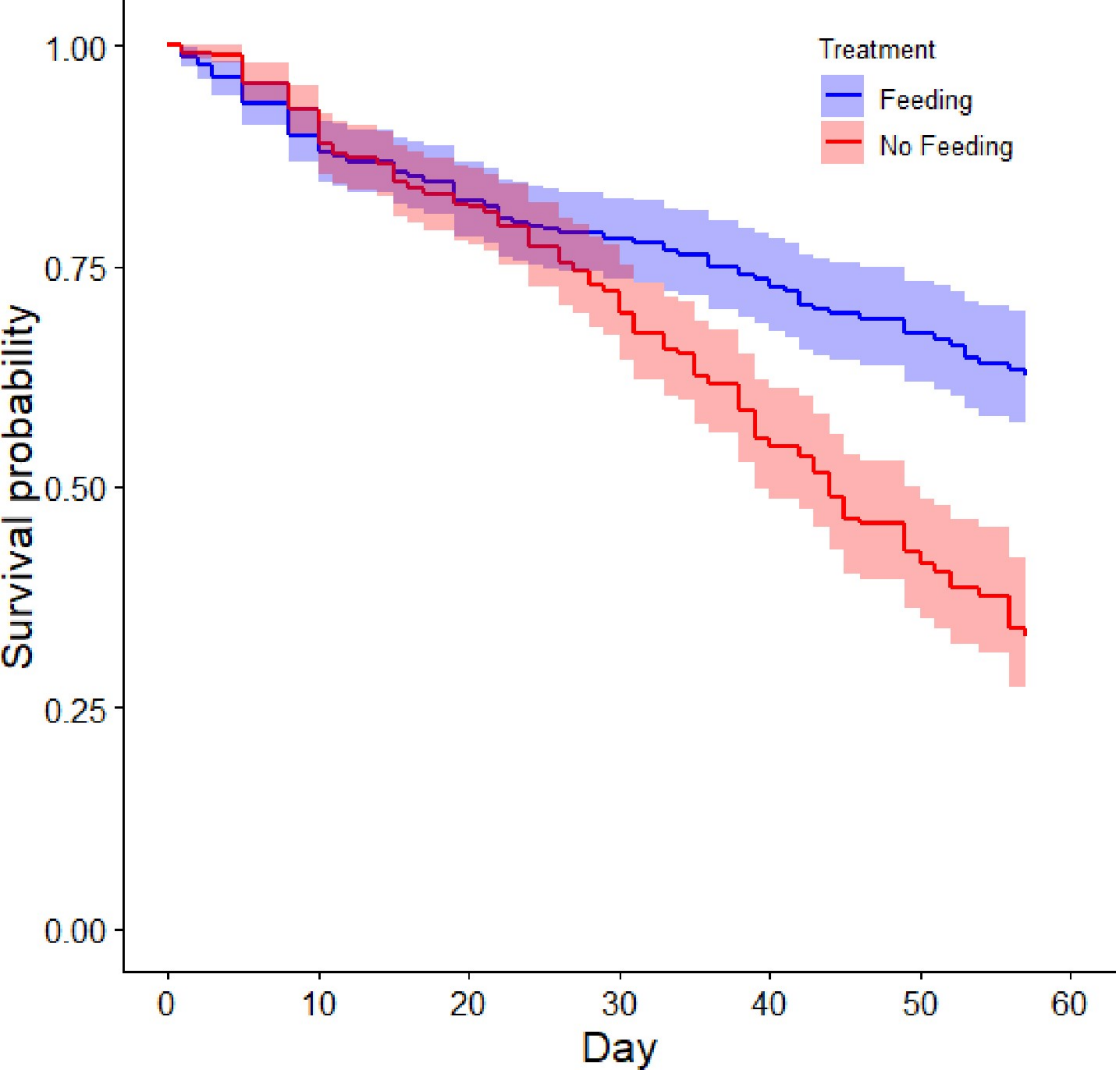
Kaplan-Meier survival probability throughout the starvation experiment. The blue line shows the Feeding treatment (control) and the red (lower) line shows the same for the No Feeding treatment. Shaded areas are 95% confidence intervals. We note that Delta Smelt is an extraordinarily delicate species [51, 53], so the mortality rate in the Feeding treatment was not atypical.

Our final analysis compared wild Delta Smelt to the hatchery-reared Delta Smelt used in our experiment. We calculated means and standard deviations of variables that were measured on both the wild and experimental fish, including condition factor, hepatosomatic index, liver lesion score, liver glycogen depletion, RNA to DNA ratio, and triglycerides in muscle. For the experimental fish, means were calculated from Day 21–56, a period for which we know starvation was severe because both hepatocyte necrosis and mortality began to increase in the No Feeding treatment 3 weeks into the experiment (Table 2, Fig 2). We excluded all fish in the wild fish dataset that were outside the body weight range of the experimental fish to improve comparability (0.085 to 1.474 g). Sample sizes for each metric are in Table 3. All relevant data are within the S1 Data.

**Table 3:** Comparison of wild Delta Smelt to hatchery Delta Smelt from the Feeding and No Feeding treatments. For the hatchery fish, means were calculated across Day 21–56 (i.e., 21 days is the point where starvation induced mortality was first apparent, Fig 2).

	Feeding			No Feeding			Wild		
Biomarker	Mean	SD	n	Mean	SD	n	Mean	SD	n
CF	0.553	0.082	96	0.418	0.074	94	0.696	0.086	967
HSI	1.67	0.607	96	0.723	0.239	93	0.793	0.287	854
Liver LS	0.25	0.532	24	2.29	1.83	24	0.380	0.915	526
Liver GD	0.417	0.717	24	2.083	0.974	24	1.854	0.993	526
RNA/DNA	1.89	0.600	96	1.35	0.45	94	1.984	0.828	665
Triglycerides	0.175	0.043	96	0.133	0.043	94	0.294	0.191	356

CF is condition factor, HSI is hepatosomatic index, Liver LS is liver lesion score, and Liver GD is liver glycogen depletion (higher scores indicate worse condition for Liver LS and GD).

## Results

### Water quality

Mean water temperature during the experiment was 15.9°C (SD = 0.47), dissolved oxygen was 9.9 mg L^-1^ (SD = 0.3), salinity was 0.3 (SD = 0.04), pH was 7.7 (SD = 0.1), total ammonia nitrogen was 0.0 mg L^-1^ (SD = 0.03), nitrate was 1.5 mg L^-1^ (SD = 0.65), and nitrite was 0.0 mg L^-1^ (SD = 0.01).

### Mortality

The mortality rate was similar between the two treatments until ~Day 21, when mortality began to increase in the No Feeding treatment (Fig 2). The survival distributions for the two treatments (Feeding and No Feeding) were statistically different (Χ^2^_1_ = 21.3, P < 0.001). Based on the overlap of the 95% confidence intervals, the difference in survival between the treatments became significant at Day 32 (Fig 2). Fish in the No Feeding treatment had a median time to mortality of 44 days, 95% CI [39.0, 49.0], while survival in the Feeding treatment did not decline below 50% during the experiment. A similar percentage of censored cases was present in the Feeding (75.3%) and No Feeding (62.0%) treatments (censored cases are individuals for which survival cannot be determined, such as individuals which survived until the experiment was terminated, or sampled fish). The number of live fish remaining in the tanks at Day 56 ranged from 17–23 in the Feeding treatment and 2–8 in the No Feeding treatment.

### Condition and hepatosomatic indices

**Fig 3:**
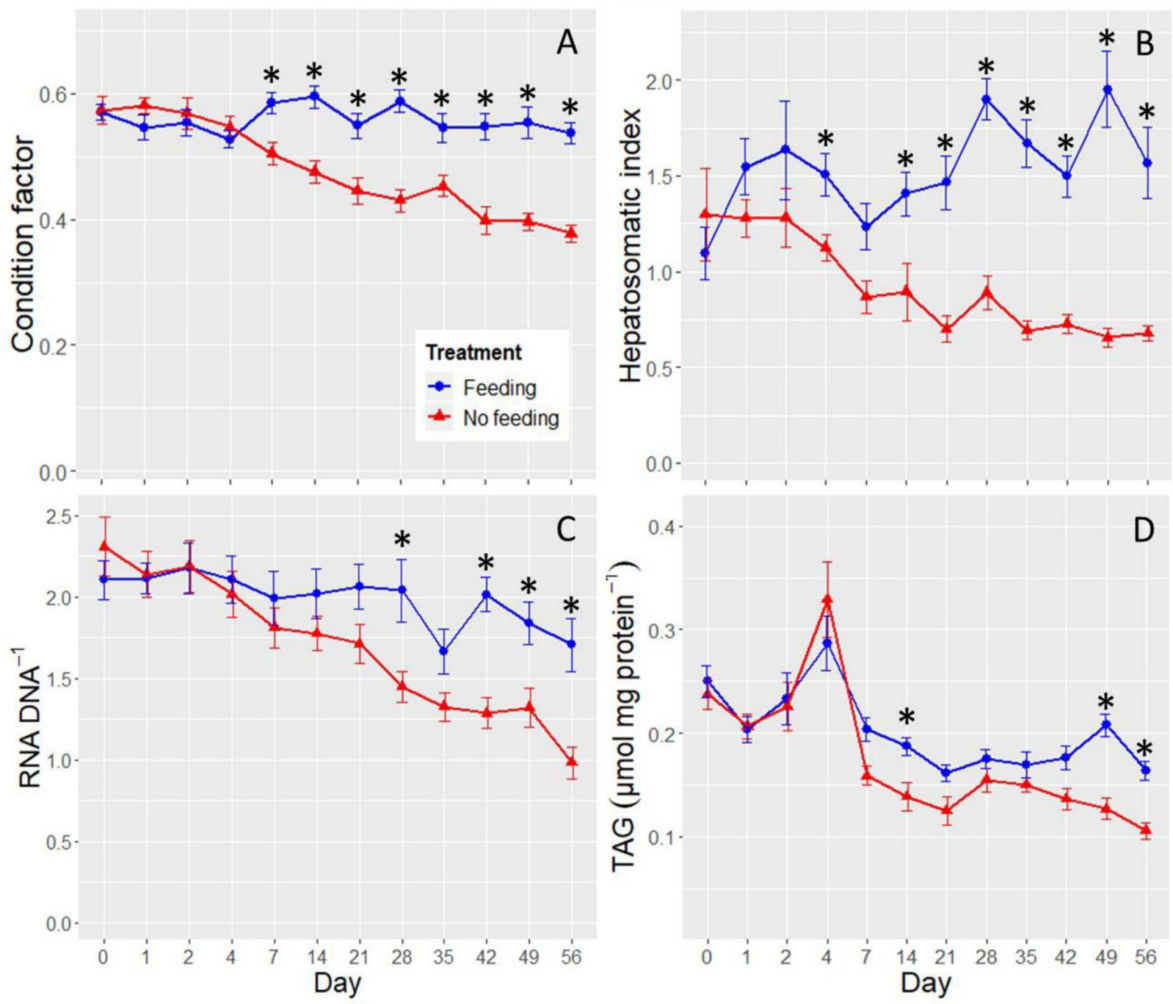
Condition factor (panel A), hepatosomatic index (panel B), RNA to DNA ratio in muscle (Panel C), and triglycerides in muscle (panel D) by day. The Feeding treatment is shown by blue circles and the No Feeding treatment by red triangles. The stars denote the time points at which significant differences were detected between the two treatments. The error bars are ±SE. The x-axis is not to scale.

Condition factor declined with an increase in the fasting period (ANOVA, day × treatment, F_11, 352.3_ = 8.2572, P <0.0001), with the No Feeding treatment becoming significantly different from the Feeding treatment at Day 7 (planned linear contrast, P = 0.0023; Fig 3A). The fish in the Feeding treatment had a mean condition factor that was 1.16-fold higher than the No Feeding treatment at Day 7. The difference increased to 1.42-fold by Day 56.

Hepatosomatic index also declined with increased fasting (ANOVA, day × treatment, F_11, 349.2_ = 5.0831, P < 0.0001), with the No Feeding treatment becoming marginally significantly different from the Feeding treatment at Day 2 (planned linear contrast, P = 0.0579), and significantly different at Day 4 (planned linear contrast, P = 0.0421). At Day 4, the Feeding treatment had a hepatosomatic index that was 1.34-fold higher than the No Feeding treatment. This difference increased to 2.31-fold by Day 56 (Fig 3B). The decline in hepatosomatic index in the No Feeding treatment plateaued after roughly Day 21 at ~0.75, indicating that livers were no longer decreasing as a proportion of body weight.

### Nutrition and growth indices in muscle

RNA to DNA ratio declined in the No Feeding treatment as the experiment progressed (ANOVA, day × treatment, F_11, 349.2_ = 2.5505, P = 0.0041; Fig 3C). The difference between the treatments first became significant at Day 28 (planned linear contrast, P = 0.0029), when the Feeding treatment had an RNA to DNA ratio that was 1.4-fold higher than the No Feeding treatment. By Day 56 that difference had increased to 1.7-fold.

Triglyceride in muscle also declined in the No Feeding treatment as the experiment progressed (ANOVA, day × treatment, F_11, 350_ = 2.1156, P = 0.0187; Fig 3D). According to the planned linear contrasts, the difference between the treatments first became significant at Day 14 (P = 0.0308), with the Feeding treatment having triglyceride concentrations that were 1.35-fold higher than the No Feeding treatment. However, it was not until Day 49 that the differences became consistently significant (i.e., linear contrasts at Day 21, 28, 35, and 42 were not significant; Fig 3D).

### Histology and immunohistochemistry

**Fig 4:**
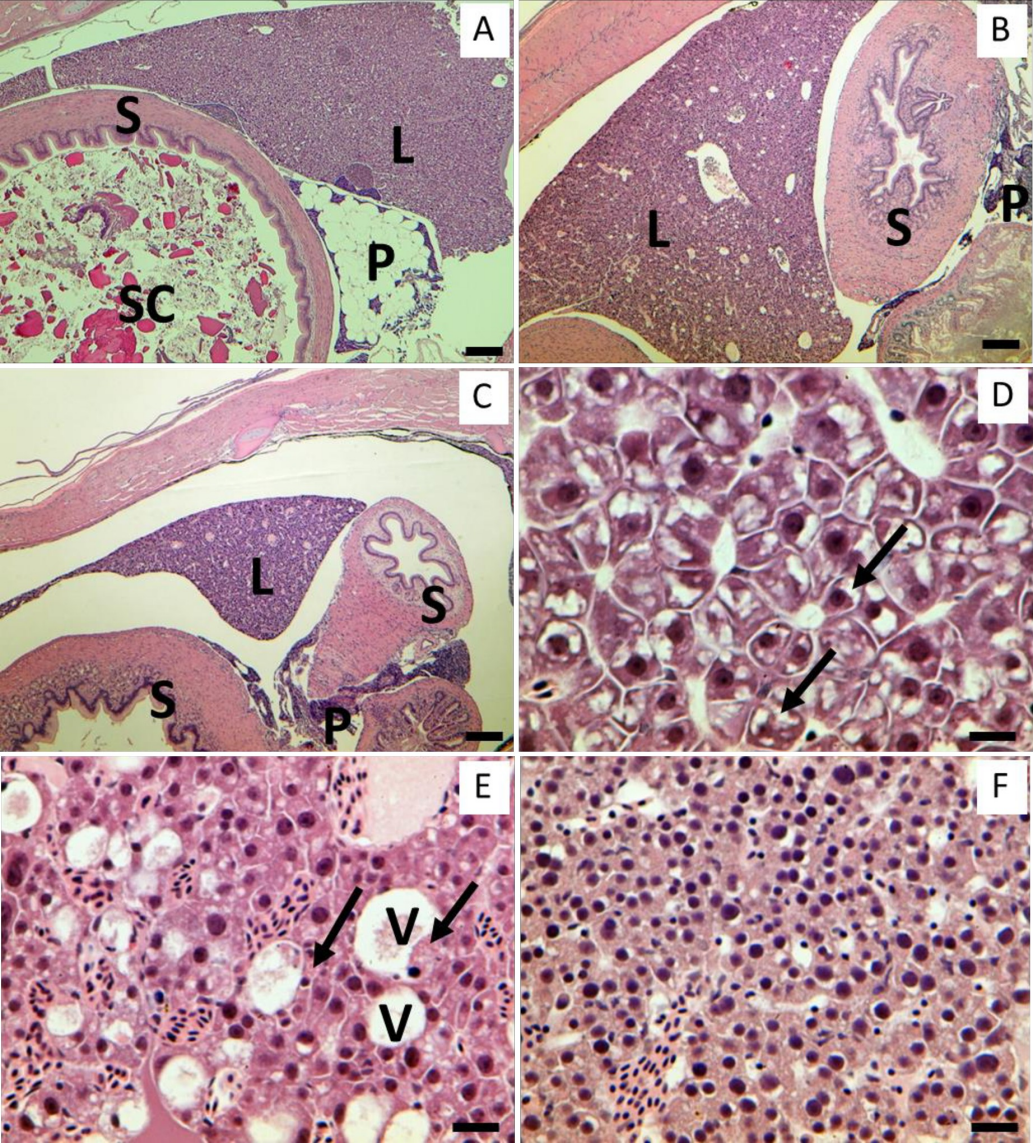
Low (Panels A-C) and high (Panels D-F) magnification starvation progression. Panel A: Delta Smelt transverse section from the Feeding treatment (control), Panel B: Delta Smelt transverse section at Day 21 from the No Feeding treatment, Panel C: Delta Smelt transverse section at Day 49 from the No Feeding treatment. S: stomach, L: liver, SC: stomach contents, P: pancreas. Note atrophy of liver, stomach and pancreas as fasting period lengthens, and empty stomach in panels B and C. Panel D: Liver of Delta Smelt from the Feeding treatment. Arrows indicate glycogen in the cytoplasm of hepatocytes. Panel E: Liver of Delta Smelt at Day 21 from the No Feeding treatment, hydropic vacuolar degeneration (V) with eosinophilic fibrillary material, likely remnants of hepatocytes. Arrows indicate necrotic hepatocytes. Panel F: Liver of Delta Smelt at Day 49 in the No Feeding treatment. Note severely atrophied hepatocytes, loss of cell-to-cell border, basophilia, and crowded nuclei. All panels H&E stained. Scale bars = 150 μm (Panels A, B and C), 15 μm (Panels D, E and F).

100% of the fish in both the Feeding and No Feeding treatments had food in their stomachs at Day 0 (8 of 8). Averaged over the rest of the time points, 90% and 10% of the fish in the Feeding and No Feeding treatments had food in their stomachs, respectively (e.g., Fig 4A and 4B). The occasional stomach contents in the No Feeding treatment appeared to be fish tissue, presumably eaten from mortalities before they were removed each day.

**Fig 5:**
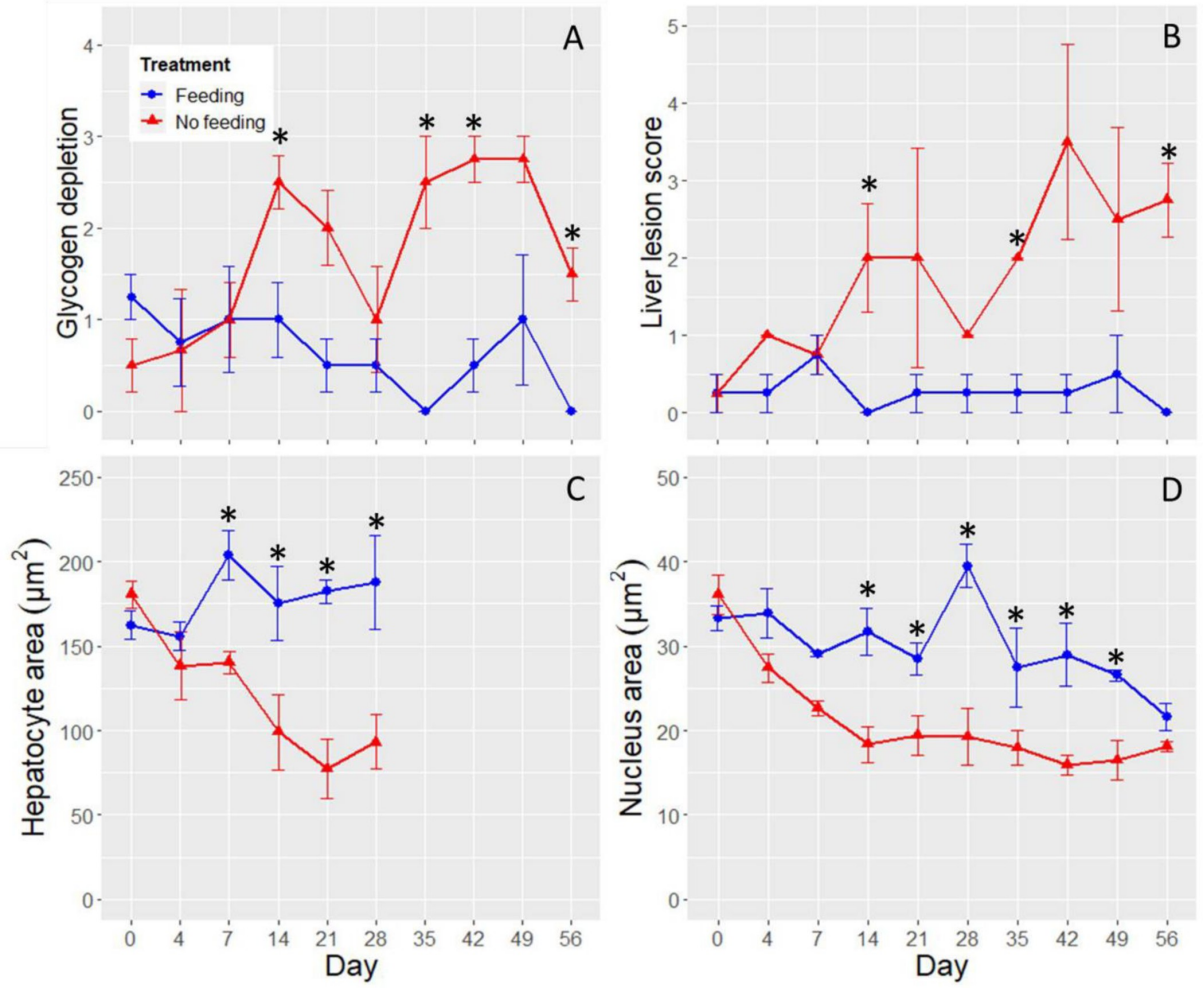
Histological indices by day. Panel A is glycogen depletion, panel B is liver lesion score, panel C is hepatocyte area, and panel D is nucleus area for the Feeding treatment (blue circles) and the No Feeding treatment (red triangles). The stars denote the time points at which significant differences were detected between the two treatments. The error bars are ±SE and the x-axis is not to scale.

A significant difference was detected between the two treatments at Day 14 for both glycogen depletion (Wilcoxon Rank Sum test, Chi Square = 4.3974, DF = 1, P = 0.0360; Fig 5A) and liver lesion score (Wilcoxon Rank Sum test, Chi Square = 6.1370, DF = 1, P = 0.0132; Fig 5B). The most prevalent lesions in the No Feeding treatment were cytoplasmic inclusion body (CIB), single cell necrosis (SCN), and hydropic vacuolar degeneration (HVD). CIB was characterized by the presence of rimmed vesicles and unknown inclusion bodies in the cytoplasm of hepatocytes (Fig 1A and 1B). Higher incidences of CIB were observed from Day 7–21 (Table 2). SCN is characterized by cells having eosinophilic cytoplasm with pyknosis and karyorrhexis. Higher incidences of SCN began in the No Feeding treatment at Day 21 and lasted through Day 56 (i.e., the rest of the experiment; Fig 4E). Higher incidences of HVD began at Day 28 in the No Feeding treatment and lasted through the end of the experiment (Day 56; Table 2, Fig 4E). Gross liver atrophy was increasingly common in the No Feeding treatment as the experiment progressed (Fig 4C).

Hepatocyte area declined as the experiment progressed in the No Feeding treatment (ANOVA, day × treatment, F_5, 28.9_ = 4.0483, P = 0.0066), with hepatocyte area becoming significantly lower in the No Feeding treatment at Day 7 (planned linear contrast, P = 0.0145; Fig 5C). At Day 7, the hepatocytes had areas that were 1.45-fold larger in the Feeding treatment. By Day 28, the last time point during which hepatocytes could be differentiated reliably in the No Feeding treatment, the hepatocytes in the Feeding treatment were 2.01-fold larger than in the No Feeding treatment (Figs 1E, 1F and 5C).

In addition to the decline in hepatocyte area, the hepatocyte nuclei became increasingly pyknotic as fasting progressed (Figs 1F, 4E and 4F). This observation is supported by the nucleus area measurements, which declined in the No Feeding treatment during the experiment (ANOVA, day × treatment, F_9, 54_ = 3.7586, P = 0.0010; Fig 5D). The difference between the treatments was marginally significant at Day 4 and 7 (P = 0.0532 and 0.0560, respectively), and highly significant by Day 14 (P < 0.0001; Fig 5D). Nucleus area at Day 14 was 1.72-fold higher in the fed treatment.

Apoptotic cells were not detected in the liver at any of the time points examined in the No Feeding treatment (Day 0 [control], 21, 42, or 56; S2 Fig).

### Oxidative stress in liver

**Fig 6:**
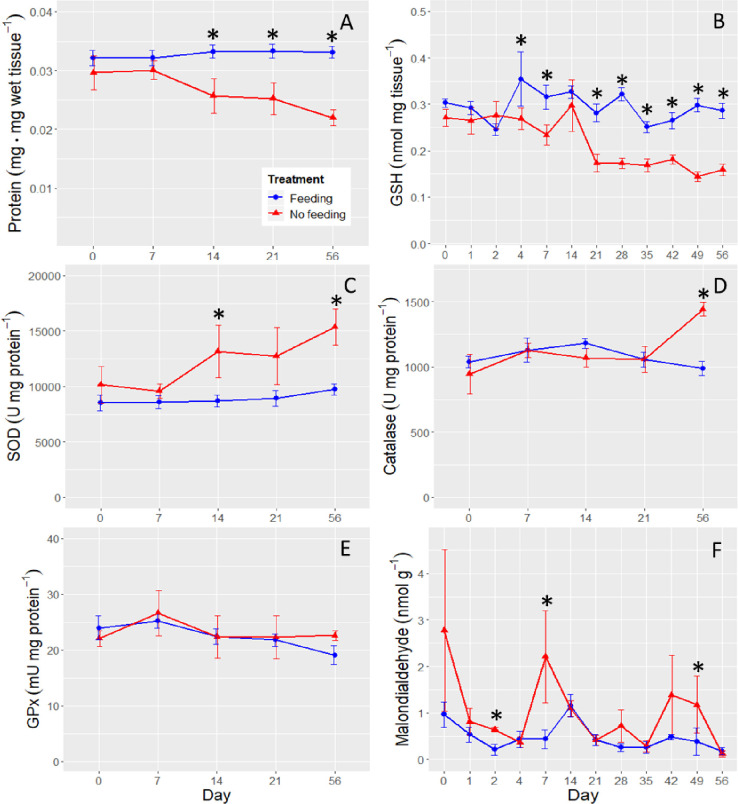
Biomarkers of oxidative stress in liver. Panel A is liver protein concentration, panel B is Glutathione, panel C is superoxide dismutase, panel D is catalase, panel E is glutathione peroxidase, and panel F is malondialdehyde. The stars denote the time points at which significant differences were detected between the two treatments. The x-axis is not to scale and the error bars are ±SE.

Protein in liver was significantly reduced by starvation (ANOVA, treatment, F_1, 29_ = 28.5707, P < 0.0001; Fig 6A). Although a day by treatment interaction was not detected (ANOVA, F_4, 29_ = 2.2552, P = 0.0875), we observed a progressive decrease in liver protein in the No Feeding treatment over time, with the difference becoming significant at Day 14 (planned linear contrast, P = 0.0068; Fig 6A). Liver protein was 1.3-fold higher in the Feeding treatment at Day 14, and reached 1.5-fold higher than the No Feeding treatment by Day 56 of the experiment.

Glutathione declined as starvation progressed (ANOVA, day × treatment, F_11, 72_ = 2.7175, P < 0.0055; Fig 6B). The difference between the two treatments first became significant at Day 4 (planned linear contrast, P = 0.0121), at which glutathione was 1.32-fold higher in the No Feeding treatment. At Day 56 the difference was 1.80-fold.

Superoxide dismutase was significantly elevated in the No Feeding treatment (ANOVA, treatment, F_1, 25_ = 15.3918, P = 0.0005), but a significant day by treatment interaction was not observed (ANOVA, day × treatment, F_4, 29_ = 1.0910, P = 0.3794). A difference between the treatments was first detected at Day 14 (planned linear contrast, P = 0.0228), when superoxide dismutase activity was 1.51-fold higher in the No Feeding treatment (Fig 6C). This difference increased to 1.58-fold by Day 56.

Catalase activity increased as the experiment progressed in the No Feeding treatment (ANOVA, day × treatment, F_4, 29_ = 4.5641, P = 0.0056), becoming significantly different at Day 56 (planned linear contrast, P = 0.0002; Fig 6D).

Glutathione peroxidase was not affected by starvation (ANOVA, treatment, F_1, 25_ = 0.2614, P = 0.6136; day × treatment, F_4, 25_ = 0.4625, P = 0.7625; Fig 6E).

Malondialdehyde was affected by starvation (ANOVA, treatment, F_1, 71_ = 7.1018, P = 0.0095), but the day by treatment interaction was insignificant (ANOVA, F_11, 71_ = 0.9367, P = 0.5111; Fig 6F). Malondialdehyde in the No Feeding treatment was 2.1-fold higher than in the Feeding treatment, averaged across all time points. While malondialdehyde became significantly different at Day 2 between the treatments (planned linear contrast, P = 0.0255), the difference was highly inconsistent over time (Fig 6F).

### Comparison to wild Delta Smelt

The wild fish were not consistently in better or worse nutritional condition compared to the experimental fish. In terms of hepatosomatic index, liver lesion score, and liver glycogen depletion, the means of the wild fish fell between the means of the fed and starved treatments (Table 3), suggesting possible nutritional stress in the wild fish. Hepatosomatic index appeared to be especially depressed in the wild fish, with very similar scores to severely starved hatchery fish. In contrast, condition factor was higher in the wild fish than even the fish in the Feeding treatment (Table 3). RNA to DNA ratio was similar between the wild fish and fed fish, and wild fish had triglycerides in muscle that were considerably higher than the fed fish. In terms of liver lesion score, wild fish had livers that were in somewhat worse condition than the fed hatchery fish, but were in far better condition than the starved fish (Table 3). In summary, the wild fish outperformed the fish in the Feeding treatment in terms of three endpoints (condition factor, RNA to DNA ratio, and triglycerides), and underperformed the Feeding treatment fish in terms of the other three (hepatosomatic index, liver lesion score, and glycogen depletion; Table 3).

## Discussion

In this study, we compared the sensitivities of an array of endpoints to increasingly severe starvation in Delta Smelt. The experiment was run at a temperature typical of fall and spring habitat of the species (15.9°C), more than ten degrees below its critical thermal maximum [40, 68, 69]. Because we ran the experiment on an ectotherm, the indices would presumably have responded more quickly if the experiment was run at a higher temperature, and less quickly if it was run at a lower temperature (e.g., [6, 22]). While there are many studies examining the responses of hatchery fish to fasting, two aspects of our study set it apart. First, a wide range of endpoints were examined, including condition, hepatosomatic, biochemical, histological, and immunohistochemical indices (Tables 1 and 2). Second, 12 time points were sampled over the two-month experiment, including multiple time points during the first week. This experimental design allowed us to compare the sensitivities of a wide range of indices to fasting, and to draw conclusions integrated across several disparate responses.

Hepatosomatic index was one of the most sensitive biomarkers measured in our study, responding significantly to fasting at Day 4 (Fig 3B). The rapid decline in hepatosomatic index indicates that the liver lost weight more quickly than extrahepatic tissue, leading to a decline in the liver’s proportion of total body weight. Hepatosomatic index is well known to decline with food limitation, occurring as hepatic metabolic reserves are consumed (i.e., glycogen, lipid, and protein), and water used to maintain osmolarity is lost [14, 15, 22, 39, 70]. Surprisingly, we did not observe an increase in liver glycogen depletion until Day 14, whereas liver glycogen typically declines within hours or days of fasting (e.g., [22, 39, 71, 72]). It is possible that the declining size of the hepatocytes made the density of glycogen appear constant on the slides during the first two weeks of the experiment, even as total liver glycogen declined (Fig 1E and 1F). A biochemical assessment of hepatic glycogen in starved Delta Smelt would therefore be necessary to confirm that liver glycogen in Delta Smelt is indeed slow to respond to starvation (e.g., [72]), precluded here due to limitation of available liver tissue.

One goal of our study was to differentiate between hepatocyte death and atrophy as mechanisms driving the rapid decline in liver weight (i.e., 4 days, Fig 3B). While a high number of apoptotic cells were present in the positive control, they were not detected in the livers of the fish from the No Feeding treatment, even after 56 days of fasting (S2 Fig). As in our experiment, Baumgarner et al. [28] detected a decline in hepatosomatic index but not apoptosis in the livers of Rainbow Trout starved for 28 days. Necrosis was detected in the No Feeding treatment, but not until the fish had been fasted for 21 days (Table 2). Thus, we found no evidence that the initial decline in hepatosomatic index was due to a decline in the number of hepatocytes. However, there was strong evidence of hepatocyte atrophy, first in terms of hepatocyte area, followed by hepatocyte nuclei area (Figs 1F, 5C and 5D). While hepatocyte area responded more slowly to fasting (7 days) than hepatosomatic index (4 days), hepatosomatic index likely had more power because it had four times the sample size (32 vs 8 fish per time point). Our interpretation is therefore that the livers lost mass largely due to hepatocyte atrophy, at least until necrosis began, at Day 21 (Table 2). This interpretation is largely in line with previous studies that show declines in hepatocyte size as starvation progresses [16, 26, 27], although Peragón et al. [14] detected a loss of DNA in the livers of starved fish, which the authors interpreted as evidence of cell loss. However, the loss of DNA occurred after 70 days of fasting at 15.0°C [14], well after necrosis became elevated in our experiment at a similar temperature (15.9°C).

Although we found that hepatosomatic index is relatively sensitive to starvation in Delta Smelt, it should not be used in isolation. In mature females, vitellogenin is produced in the liver, increasing the weight of the organ (e.g., [73]). The endpoint can also be affected by contaminants (e.g., [74, 75]), known stressors in the SFE (e.g., [46, 76, 77]). In addition, hepatosomatic index is unlikely to be as sensitive as stomach fullness, which reflects daily foraging success in Delta Smelt (e.g., [44, 78]). A suite of laboratory-validated biomarkers is therefore recommended.

The liver histology results revealed a clear progression of pathologies as starvation increased in severity. Cytoplasmic inclusion bodies, which we suspect were autophagosomes, were the first abnormality detected histologically, becoming elevated in the No Feeding treatment at Day 7 (Table 2; [79]). Autophagy is a ubiquitous, highly conserved, protective mechanism that allows cells to maintain homeostasis during exposure to a variety of stressors, including starvation, infections, and contaminants [80–82]. During autophagy, long-lived proteins and organelles are packed inside vesicles within the cytoplasm. These vesicles then fuse their outer membranes with lysosomes to form an autophagosome, which digests the enclosed material, providing nutrition [81]. Autophagy can begin 1–14 days after fasting begins, depending on the taxon (e.g., nematode: [83], fish [autophagy gene expression]: [84], mice: [85]). This timing is consistent with the appearance of cytoplasmic inclusion bodies in our experiment (Day 4–7). Starting at Day 21, the cytoplasmic inclusion bodies largely disappeared, and single cell necrosis, hydropic vacuolar degeneration, and mortality all became elevated above background levels (Table 2, Fig 2). We suggest that after 21 days of starvation, the role of autophagy as a mechanism to extend cell survival was impaired or no longer functional, leading to a failure of homeostatic mechanisms (i.e., ATP depletion and calcium homeostasis failure), necrosis, and mortality (Table 2, Fig 2). The liver protein data are also consistent with this interpretation, because liver protein became significantly depressed following the appearance of cytoplasmic inclusion bodies (Fig 6A). However, additional work is needed to confirm that the hepatic cytoplasmic inclusion bodies are autophagosomes (e.g., electron microscopy, [86]).

Another goal of our study was to distinguish liver abnormalities caused by starvation from other causes, because Delta Smelt are exposed to a variety of stressors in the SFE ([42, 87]). In Hammock et al. [44], we reported elevated lipidosis and inflammation in livers of juvenile Delta Smelt collected from Cache Slough, common responses of fish to contaminants (e.g., [76, 88, 89]). In the present study, liver lipidosis and inflammation were largely absent, even in severely starved fish. Instead, the major lesions were cytoplasmic inclusion bodies, followed by cell necrosis and hydropic vacuolar degeneration (Table 2). Thus, although the starved fish had unhealthy livers, like wild fish collected from Cache Slough, the specific lesions were quite distinct. Moreover, other lines of evidence support the hypothesis that the livers of Delta Smelt collected from Cache Slough were damaged by contaminants rather than nutritional stress, including: 1) both zooplankton abundance and stomach fullness are relatively high in freshwater in summer [44, 45], 2) Delta Smelt collected from Cache Slough exhibited robust condition relative to fish collected from other regions [44], 3) contaminants have occurred in relatively high concentrations in the region [47], and 4) acute toxicity has been repeatedly detected in the region [46, 48, 77]. In addition, parasites and bacterial infections are unlikely causes of lesions because they are rare in the wild Delta Smelt population ([90], S. Teh *personal observation*), as well as the hatchery Delta Smelt. Thus, the present results strengthen the association between contaminants and liver alterations previously observed in Delta Smelt collected from Cache Slough [44], and will help disentangle liver damage caused by contaminants from nutritional stress in future studies.

RNA to DNA ratio and triglycerides in muscle were relatively insensitive to starvation, responding significantly at Day 28 and 14, respectively (Fig 3C and 3D). The RNA to DNA result was surprising given that the endpoint generally responds rapidly to changes in feeding rate. In one review, Buckley et al. [18] reported that RNA to DNA ratio responds to food limitation in as little as 1–3 days in a variety of young fishes. More recently, Duguid et al. [91] detected a decrease in RNA to DNA ratio within 6 days after a reduction in feeding in juvenile Atlantic Salmon. While triglycerides in muscle responded more quickly than RNA to DNA ratio, the difference between the Feeding and No Feeding treatments was small and inconsistent during the remaining six weeks of the experiment (Fig 3D). Thus, neither RNA to DNA ratio or triglycerides in muscle consistently responded to starvation before mortality rate became elevated (Fig 2). This presents a problem for detecting food limitation using these metrics in the wild, particularly if sample size is low, because Delta Smelt may experience lethal levels of nutritional stress before a response in either metric is detectable. One possible solution would be to quantify RNA to DNA ratio and triglycerides in liver tissue, which was highly sensitive to starvation in other ways (Figs 1, 4 and 5). In Zebrafish for example, Lu et al. [72] recently detected a decline in triglycerides in liver after 3 days of fasting, at 28°C.

One difficulty associated with measuring multiple endpoints on wild fish is that they can disagree, complicating interpretation. Hammock et al. [44] reported significantly depressed stomach fullness, condition factor, RNA to DNA ratio, and hepatosomatic index in Suisun Bay, a region that has exhibited a crash in primary and secondary productivity (e.g., [2, 92, 93]). However, the biomarker data were not entirely consistent with the paper’s conclusion of nutritional stress in the region, because triglyceride concentration in muscle, 10-day otolith increment, and glycogen depletion were not significantly depressed in Suisun Bay [44]. Our rationale was that 10-day otolith increment and triglycerides in muscle might have been homogenized by movement of individuals among regions before they could respond to the environmental conditions of the region, whereas biomarkers that respond more quickly to environmental conditions, like stomach fullness, may better reflect conditions where an individual was collected [17, 18, 44]. However, the lack of a response in glycogen depletion weakened this interpretation because it generally responds rapidly to food limitation (e.g., [15, 22]). The current study therefore helps reconcile this inconsistency, demonstrating that glycogen depletion, at least when estimated histologically (as was the case in Hammock et al. [44]), is relatively slow to respond to food limitation in Delta Smelt. Thus, our experimental results strengthen the conclusion of summertime nutritional stress for Delta Smelt in Suisun Bay [44].

Of the four antioxidants measured, one decreased (glutathione), two increased (superoxide dismutase and catalase), and one was not affected by starvation (glutathione peroxidase; Fig 6). The net result was a roughly two-fold increase in levels of lipid peroxidation in the No Feeding treatment that was highly inconsistent through time (Fig 6F). This contrasts with other fish species which show more consistent starvation-induced oxidative stress responses due to inadequate neutralization of reactive oxygen species [32, 33, 39, 94]. Our interpretation is that the increase in activities of catalase and superoxide dismutase could not compensate for the decreased concentration of glutathione, leading to an overall increase in lipid peroxidation in the starved fish. Another contributing factor to pro-oxidant effects of starvation may have been the loss of food-derived antioxidants such as vitamins C and E [94]. Given the evidence for both food limitation [41–43, 45]) and contaminants [46–48, 76, 77] in its habitat, Delta Smelt in the wild may be vulnerable to oxidative damage caused by contaminants, especially xenobiotic electrophiles detoxified by glutathione (e.g., organophosphates, such as chlorpyrifos or polycyclic aromatic hydrocarbons). Future work could explore the potential interaction between starvation and xenobiotics that are detoxified by glutathione-dependent mechanisms.

The comparison of nutritional condition between fed, starved, and wild Delta Smelt was inconclusive. On one hand, hepatosomatic index and glycogen depletion of the wild fish were very similar to the severely starved experimental fish, suggestive of nutritional stress in wild Delta Smelt (Table 3). On the other hand, condition factor, RNA to DNA ratio, and triglycerides were higher in wild fish than even the control fish, suggesting the opposite (Table 3). This result is unsurprising given that stark differences between hatchery and wild fish, especially in terms of diet and environmental conditions, are known to make comparisons difficult [21]. We note that although triglycerides in muscle were higher in the wild fish, there was far more adipose tissue present in the abdomen of the control hatchery fish (S. Teh, *personal observation*), which is typical of hatchery fishes [95]. Therefore, triglyceride in muscle appears to be a poor indicator of lipid reserves in Delta Smelt. Overall, we consider the relative sensitivities and mechanisms of the responses to fasting as more applicable to the wild population than the values of the biomarkers themselves.

In conclusion, despite extensive work on other fishes, species-specific validation of biomarkers is necessary. In Delta Smelt, hepatosomatic index was highly sensitive to starvation, condition factor was somewhat sensitive, and RNA to DNA ratio in muscle, triglycerides in muscle, and glycogen depletion in liver (estimated histologically) were relatively insensitive. The initial decline in hepatosomatic index was most likely due to hepatocyte atrophy, rather than apoptosis or necrosis. In terms of liver histology, starved fish first exhibited cytoplasmic inclusion bodies, followed by glycogen depletion, necrosis, and hydropic vacuolar degeneration. Importantly, we detected no inflammation or lipidosis associated with starvation, in contrast to wild fish collected from Cache Slough [44]. Starvation induced a mixed response in hepatic antioxidants, resulting in lipid peroxidation. Glutathione in particular was rapidly lost as starvation progressed, suggesting that Delta Smelt under nutritional stress may become vulnerable to electrophilic contaminants. While some biomarkers were more sensitive to starvation than others, a range of endpoints are still recommended for use in wild fish exposed to multiple stressors. These could include not only a variety of biomarkers on the focal species, but also environmental data, such as toxicity, analytical chemistry, and prey abundance. Overall, our results strengthened the conclusions of Hammock et al. [44] of regionally specific stressors.

## Supporting information

S1 Fig Experimental tanks. (DOCX)

S2 Fig Apoptosis results. (DOCX)

S1 Data (XLSX)
