# Supplementary material for: The health and condition responses of Delta Smelt to fasting: A time series experiment
Source: PLoS One. 2020 Sep 24;15(9):e0239358. doi: 10.1371/journal.pone.0239358 (PMC7514091; doi:10.1371/journal.pone.0239358)
Supplement: S1 Fig — (DOCX) [file pone.0239358.s001.docx]

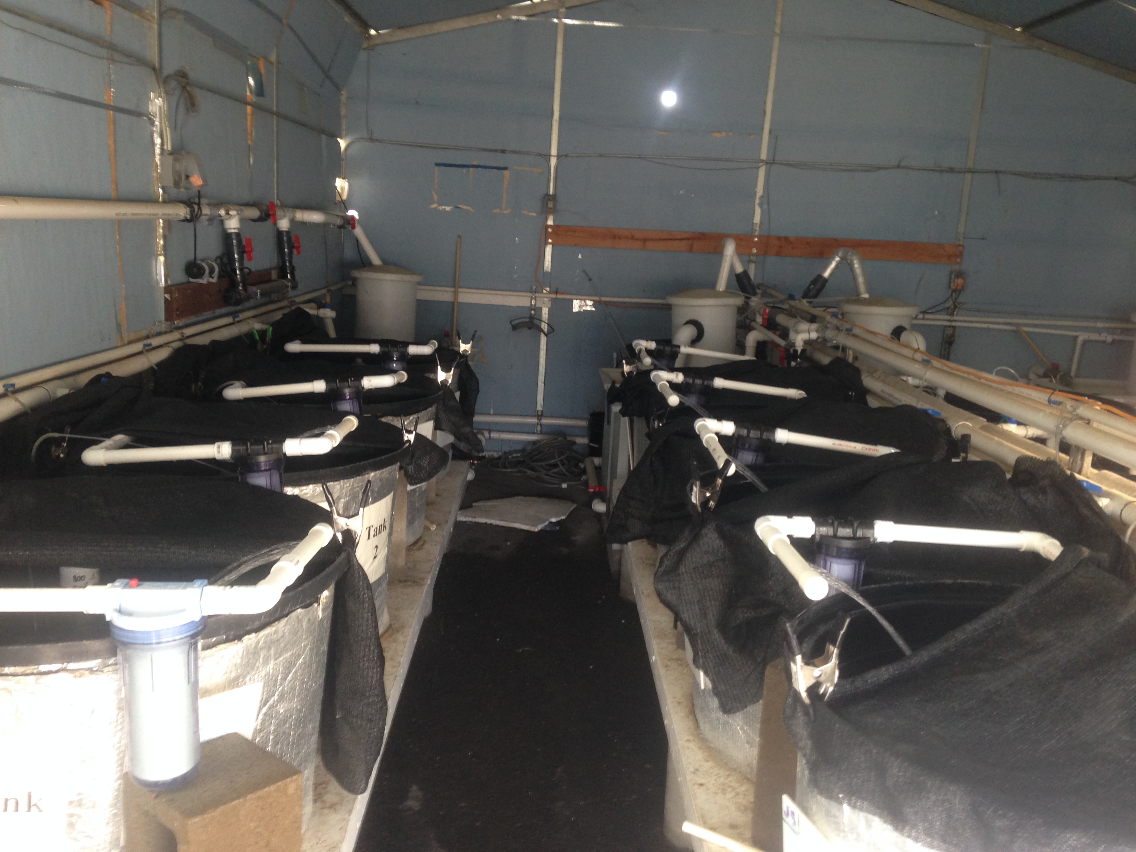


Fig. S1 Tanks used in the starvation experiment. Each tank was equipped with a 5 µm filter on the input to ensure that no particles of food reached the No feeding tanks. Each tank also has black shade-cloth to minimize light. The treatments were randomly assigned to each tank (4 tanks for each treatment).
