# Supplementary material for: The health and condition responses of Delta Smelt to fasting: A time series experiment
Source: PLoS One. 2020 Sep 24;15(9):e0239358. doi: 10.1371/journal.pone.0239358 (PMC7514091; doi:10.1371/journal.pone.0239358)
Supplement: S2 Fig — (DOCX) [file pone.0239358.s002.docx]

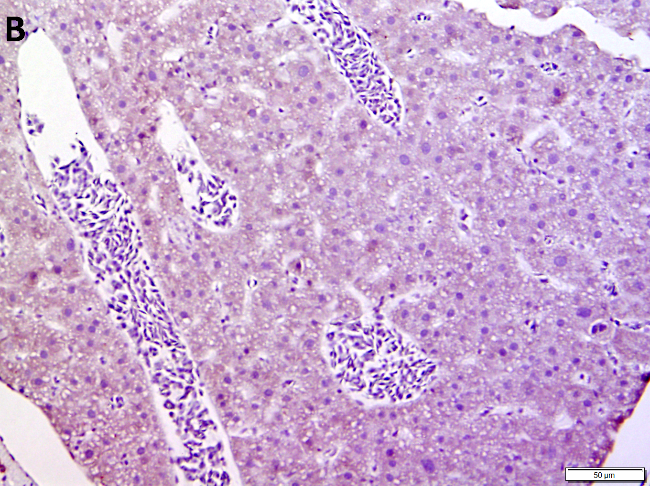

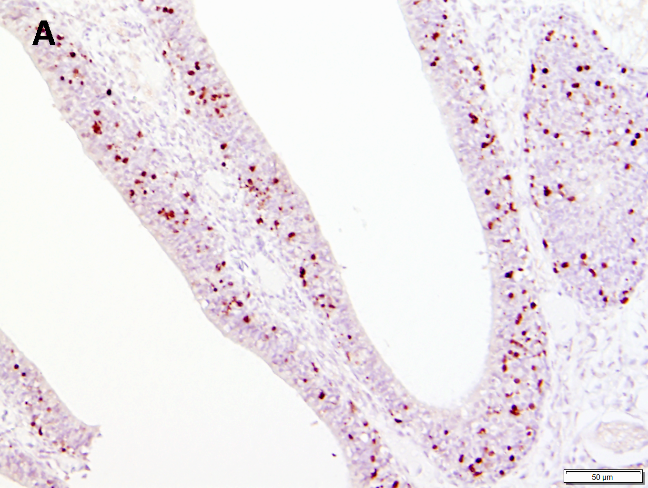

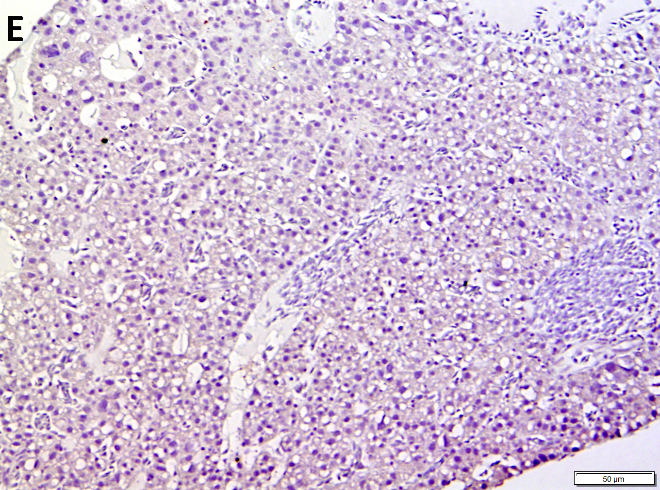

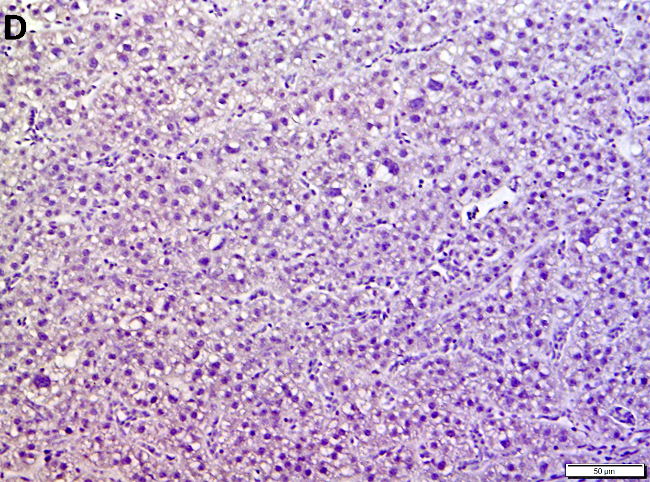

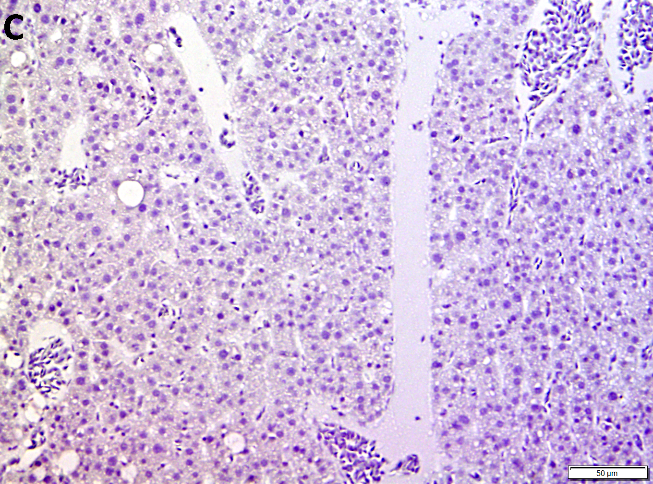


Fig. S2 Panel A: Anti-Caspase 3 Immunohistochemistry of olfactory epithelium from copper-exposed Delta Smelt used as a positive control. Multiple caspase 3 positive apoptotic cells are demonstrated (red-brown staining). Panels B, C, D and E: Anti-Caspase 3 immunohistochemistry of liver from the 0 day time-point (control, panel B) and fish starved for 21 days (panel C), 42 days (panel D), and 56 days (panel E). Magnification 200×
